# The Cranial Bowl in the New Millennium and Sutherland's Legacy for Osteopathic Medicine: Part 1

**DOI:** 10.7759/cureus.10410

**Published:** 2020-09-12

**Authors:** Bruno Bordoni, Stevan Walkowski, Bruno Ducoux, Filippo Tobbi

**Affiliations:** 1 Physical Medicine and Rehabilitation, Foundation Don Carlo Gnocchi, Milan, ITA; 2 Osteopathic Manipulative Medicine, Heritage College of Osteopathic Medicine-Dublin, Ohio, USA; 3 Osteopathy, Formation Recherche Osteopathie Prévention, Bordeaux, FRA; 4 Osteopathy, Poliambulatorio Medico e Odontoiatrico, Varese, ITA

**Keywords:** osteopathic, cranio, fascia, craniosacral, diaphragm

## Abstract

A theoretical model that does not evolve with new information deriving from scientific research, by changing the assumptions from which it was born, becomes a philosophy; the scientist becomes a scholarch. Cranial manual osteopathic medicine is very controversial, although it is commonly practiced, from the clinician to the nonmedical health worker. The article, divided into two parts, reviews the assumptions with which the cranial model was created, highlighting the scientific innovations and new anatomical-physiological reflections. In the first part we will review the synthesis and movement of cerebrospinal fluid (CSF), the movement of the central and peripheral nervous system; we will highlight the mechanical characteristics of the meninges. The aim of the article is to highlight the need to renew the existing cranial model.

## Introduction and background

The osteopathic cranial manipulative medicine (OCMM) was born around 1898, when a student of the American School of Osteopathy observed a disjointed skull in the museum of the same institute and noticed that the temporal bones resembled the gills of fish [1]. The osteopathic student saw that the bone contours were built to join other bones as a complex joint. The similarity with the gills and the bone shape allowing the articulations of the skull, gave the inspiration for the fundamental principles of OCMM: the primary respiratory mechanism (PRM) and the movement of the cranial bones [1]. The student was the future Dr William Garner Sutherland DO. The PRM is the theory that would explain the movement of the cranial bones through palpation [2]. This theory is based on five principles: the fluctuation of the cerebrospinal fluid (CSF); the inherent motility of the central nervous system and spinal cord; the mobility of the meningeal membranes (cranial and spinal); joint mobility of the bones of the skull; the involuntary (passive) movement of the sacrum between the iliac bones [3]. Also according to this theory, the skull has a cranial rhythm or cranial respiration; this rhythm has an palpatory oscillation of about 12 cycles per minute [3]. Another cornerstone of PRM is the movement of the joint between the occipital bone and the sphenoid bone (spheno-basilar synchondrosis or SBS), an important fulcrum for identifying structural dysfunctions of the shape and function of the cranium [3]. According to OCMM, the midline bones include the sphenoid bone, occiput, ethmoid, and vomer. During PRM of the cranium, these would undergo flexion and extension and would be responsible for the external and internal rotation movement of the other paired cranial bones, respectively [3]. During the flexion of the SBS, the meninges pull the sacral bone upwards, resulting in an anatomical extension or counternutation of the sacral base; the opposite happens during an extension of the SBS [3]. The osteopath places his hands on the skull and evaluates any cranial dysfunctions [2]. A cranial dysfunction refers to a perceived movement that is foreign to what should be normal (flexion-extension or internal-external rotation) [1]. Classically, with palpation and following PRM theory, various specific cranial lesions can be identified at the SBS: shears, lateral strains, torsions, sidebending rotations and compressions, among others [3]. Other cranial lesions that can be palpated are attributable to dural dysfunction, such as lateral (right or left) and upper or lower strains; additional lesion include intraosseous strains and abnormal movements of individual skull bones (29 bones in adults) [3-4]. From the Foundation of Osteopathic Research and Clinical Endorsement (FORCE) numerous publications on fascial tissue, a study group is born to understand, improve, and possibly create a new model of OCMM [5-6]. This group, Cranial Research And New International Osteopathy: CRANIO, involves osteopaths and researchers of different nationalities. The work we propose is divided into two articles. The first part aims to review and compare the most recent scientific knowledge with respect to the theoretical basis on which the PRM in adults is based, trying to highlight the need for a new model of OCMM. The second part will complete the review, and will try to propose a new way of conceiving OCMM, again through the current scientific literature.

## Review

Production of CSF

According to OCMM, cranial movement begins with the synthesis and distribution of CSF [7-8]. The presence of fluid surrounding the cortex has been known since the time of Imhotep, an Egyptian physician of 4000 years ago, and from Herophilus (280 BC) and Hippocrates (370 BC), as well as Galen of Pergamon (200/216 AD) [9]. Hippocrates was the first to describe this fluid as water that surrounds the brain, until 1764 the physician Italian Cotugno, who described the presence of water but giving a specific name: cotunnii CSF [10-11]. The terminology used today, namely CSF, appeared only in 1842 in the writings of Magendie [11]. CSF has two ontogenic origins, depending on the stage of development of the embryo. The neural plate, which derives from a portion of the dorsal ectoderm, is in contact with the amniotic fluid (AF); the neural plate bends to form the neural tube with a longitudinal vector [12]. This movement traps part of the AF inside the neural tube. The primitive neural tube is in contact with the AF, thanks to the opening of the neuropores, which are not yet closed; this means that its development is affected by the fluidic information of the AF [12]. The neural tube undergoes other transformations. In its posterior portion it will form the future spinal cord while, in the anterior area it will expand to form the central nervous system [12]. The neurogenic placodes will remain in contact with the AF while the nascent brain system will come into contact with the fluid that will become the CSF, passing through the AF [13]. In the early stages of brain formation, the latter is an empty space filled with fluids and surrounded by a primitive mono-layered neuroepithelium. When the anterior neuropores close permanently, the brain area has independence in the production of CSF and no longer reflects the metabolic changes of the external fluid environment [12]. The precursor layer of neuroepithelium develops further, following a specific histogenesis. During embryogenesis, CSF does not circulate but instead facilitates the swelling and enlargement of the spaces that will be filled by the structures of the nervous system; the capillaries, then play a fundamental role in the CSF secretion and resorption [14]. During this period of formation, the mesodermal system produces the CSF (blood vessels). When the embryonic period ends, that is, with the appearance of the sketches of the choroid plexuses, the CSF is produced by ectodermal structures [15]. The choroid plexuses become an important CSF production center. Neurogenesis begins when the plexuses begin to work [15]. The molecules present within the CSF of the fetus and the adult are different with different functions [15]. Until the appearance of the plexuses (41 days of gestation) in the fourth ventricle, CSF does not circulate [15]. Another difference of fetal CSF is that the latter acts to create expansive forces, while that of the adult is to keep fluidic forces in balance [15]. One of the key points of cranial rhythm is the production of CSF from the choroid plexuses (from blood plasma), and then released into the ventricles. In reality, there are other sites of synthesis, such as the circumventricular organs, the neuroepithelial layer that covers the ventricles, albeit to a lesser extent [9]. The average daily production of CSF is 400-600 mL, with a constant volume of about 150 mL and an average pressure in adults of about 10-15 mmHg; production is influenced by various factors, including the innervation of the choroid plexuses [14]. The sympathetic system tends to reduce the secretion of CSF while the parasympathetic stimulus stimulates the synthesis of this fluid [14]. Some neuropeptides (with specific receptors on the plexuses), have been shown to play a role in production, such as serotonin, dopamine, melatonin, as well as atrial natriuretic peptide (ANP) and arginine vasopressin (AVP) [14]. ANP is secreted by myocytes in the atria of the myocardium, in particular, with the main objective of reducing blood pressures, while at the level of the plexuses, by activating the aquaporin-1 channel (AQP1), it is able to reduce the synthesis of CSF [16]. Heart disease, such as chronic heart failure (CHF) could adversely affect CSF production. AVP is synthesized in some brain areas and by the epithelium of the choroidal plexuses, in particular when there is an alteration in the amount of water in the body, involving the voltage-gated Na (+) channels and specific receptors such as AVP V1 receptor; its action on the plexuses is to stimulate the reduction of fluid production [17]. Considering that AVP is also known as a "social" molecule, mood swings could induce changes in fluid production. According to some researches, the brain parenchyma is another production resource, especially due to the contribution of water [18]. An important scientific information on the behavior of CSF is the variation of the production area, depending on the person's posture. It has been shown that if the person is supine, the synthesis comes in particular from the ependymal cells of the spinal cord while, if the person is erect, the CSF synthesis occurs mainly from the choroid plexuses [19-20]. CSF synthesis does not occur only in the ventricles but also in other anatomical areas; the synthesis depends on the posture and on different molecules and nervous systems that influence the behavior of the choroid plexuses. It depends on the presence of light and dark (circadian rhythm). Melatonin, in addition to the production by the pinealocytes (which are in contact with the CSF), is produced by tanycytes, specialized ependymal cells that are found in the third ventricle; melatonin synthesized at night seems to have the ability to increase the synthesis of CSF, from 12 mL per hour during the day, up to a maximum of 42 mL per hour at night [19, 21-22]. CSF is also synthesized by the brain parenchyma and the pia mater of the brain surface [23]. The synthesis of CSF is not a passive process but is the response to multiple variables for homeostatic control, with respect to the environment in which it circulates, and with respect to environmental variables.

Movement of the CSF

**Figure 1:**
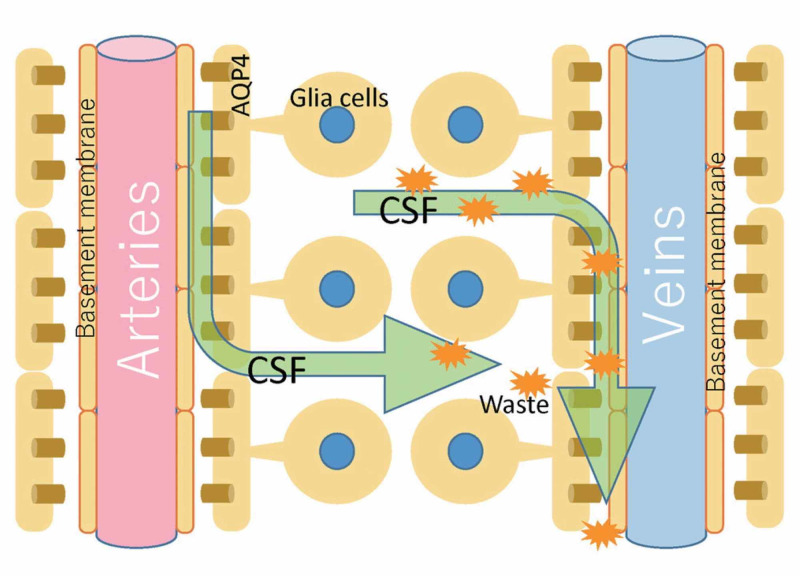
The figure illustrates schematically what the distribution of CSF could be through the perivascular spaces, in admixture with the interstitial fluids. CSF, cerebrospinal fluid Image reproduced with permission of Toshiaki Taoka, MD, PhD and colleagues, Department of Innovative Biomedical Visualization, Nagoya, Japan.

Another concept that creates a dichotomy with the osteopathic model of PRM concerns cerebral and spinal fluid motion. The circulation of the CSF is not unidirectional and its flow (direction, force, volume, speed) is not uniform. The flow does not correspond to the amount of CSF produced, and can be bi-directional and oscillatory [24]. We could compare its flow as random and heterogeneous as a Pollock painting. In the central and spinal nervous system, although we know little and there are studies on animals and little on humans, the displacement of CSF is also stimulated by some structures referred to as Reissner fibers (RFs). RFs consist of the aggregation of a glycoprotein (scospondin) and, although they play an important role during embryogenesis, some evidence highlights the role in facilitating the flow of fluid; in adults, probably, they would have a role of mechanotransductive sensor at the passage of CSF [25-26]. The absorption of CSF through the arachnoidal villi and the outflow to the venous system, as generally taught in the cranial osteopathic setting, is not so true [23]. Under physiological conditions, the most important route of circulation for CSF to exit the skull is the nasal lymphatic system [23]. The perivascular space that surrounds the veins and arteries at the subarachnoid level, and which penetrates and surrounds the brain parenchyma, is known as the Virchow-Robin space [10]. The mechanisms that allow the movement of CSF in this space are linked to arterial pulsations and thanks to a mechanism known as convection; the latter is the motion of fluids through the relationship between the heat of the fluid itself and its mass gradient (Figure 1) [27].

 

**Figure 2:**
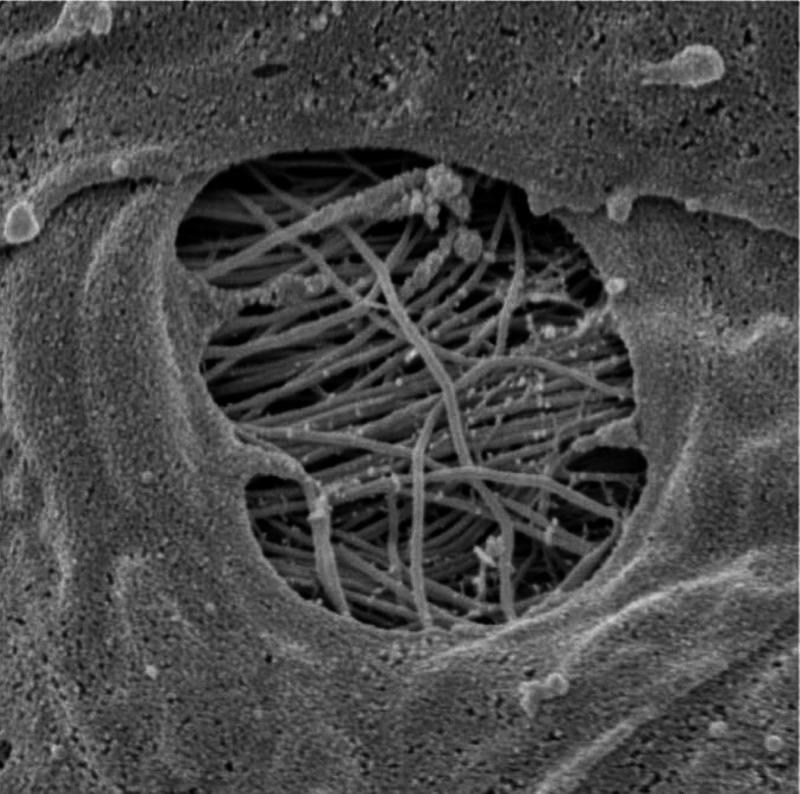
The image shows a pore or stomata of a vessel in the subarachnoid space in which the CSF can penetrate into the perivascular space. CSF, cerebrospinal fluid Image reproduced with permission of Prof. Joan Abbott, Faculty of Life Sciences and Medicine, Institute of Pharmaceutical Science, King’s College, London, UK.

Furthermore, the CSF can penetrate the perivascular space thanks to the pores of the vessels (in particular at the level of the subarachnoid space), called stomata (Figure 2) [21].

The CSF, having penetrated into the arterial perivascular space, enters the cerebral interstitium, thanks to the regulation of aquaporin proteins (aquaporin 4 - AQP4) of the water channels (CSF is 99% water); in these channels are found the terminal portions of the astrocytes or glial cells, which constitute the outer wall of the perivascular space [23]. In this process, CSF mixes with the interstitial space, exchanging solutes (with diffusion mechanisms), molecules and cleaning the different metabolic residues [23]. Once the interstitial fluid is cleansed, the CSF travels to the venous perivascular space [23]. The exchange mechanism between interstitial fluids and CSF at the level of the perivascular spaces is defined as the glymphatic system (glial cells and lymph) [21]. We know that there are meningeal lymphatic vessels but, probably, they do not have direct exchanges with CSF, if not out of the nervous system [28]. The pathways that CSF follows while exiting the skull are varied. From the venous perivascular space, the CSF reaches the olfactory bulb, to enter the perineural space of the olfactory nerve (15%-20% of all the CSF), up to the nasal mucosa, where the CSF will be drained by the lymphatic vessels of the mucosa; from here, the fluid enters the sub-buccal and mandibular lymphatic tracts, up to the cervical and spinal lymphatic nodes [28]. At the base of the skull there are many lymphatic vessels that penetrate the skull, following the exit routes of the cranial and spinal nerves [11]. Probably, the CSF has the possibility to enter these peripheral vessels and, again, to enter the lymphatic lymph nodes of the cervical tract, also following the spinal pathways [11]. CSF is also absorbed by the inner ear, the internal carotid, the perineural space of the cranial nerves, spinal and intercostal nerves, and the cranial venous system [11, 21]. Probably, the CSF that follows the perineural space towards the muscle tissue, could enter in contact with muscle interstitial fluids.

Pulsation of the CSF

The major forces that are documented to determine the movement of the CSF are the heartbeat and diaphragmatic breathing; the heart and the diaphragm muscle create the pulsations of the CSF. During an inhalation, the CSF is pushed cranially (from the perivascular and perineural spinal space towards the skull), while with an exhalation, the movement is pushed caudally [19]. The intracranial pressure of CSF in the supine position is the lowest (4.6 mmHg) compared to other postures and despite the centripetal and centrifugal forces [19]. Probably, the breath acts primarily on the perivascular venous pathway but more in-depth studies on humans are lacking [29]. The systole and diastole are able to move the CSF, albeit with less emphasis than the diaphragm muscle. Probably, the heartbeat would most influence movement in the arterial perivascular space [29]. During systole, the CSF is pushed caudally, while, with diastole, the CSF is pushed cranially. It should be remembered that the movement of CSF, thanks to the breath and the rhythmic beating of the heart, is never complete throughout the body (it does not make a complete turn); the movement itself stimulates waves, rhythms which allow the CSF to move within the body system [24]. The unidirectionality of the CSF does not exist while the pulsations of the CSF are found throughout the body [24]. One structure that could act as an aid in the bi-directional displacement of CSF between the base of the skull and the cervical spinal cord is a lamina of arachnoid tissue with a rhomboid morphology, between the medulla oblongata and the cervical roots (C4) [30]. It is called "Valva Cerebri" for its hydrodynamic role for the movement of the CSF; further studies are needed [30].

The inherent motility of the central nervous system and spinal cord

According to the concept of OCMM, the central and peripheral nervous system would have an intrinsic or inherent movement, capable of moving the CSF and the cranial meninges [1-3]. These movements would determine the rhythm of the PRM and, consequently, the rhythm felt by the palpatory evaluation of the osteopath (10-14 cycles) [8]. There are many elements and forces capable of influencing the motility of the nervous system. The extracellular matrix that pervades the brain and spinal cord (and the whole body) has piezoelectric properties. An electrical stimulus traveling through the matrix will cause a vibration, while the passage of mechanical-metabolic information will cause the formation of electricity [31]. These changes in the behavior of the extracellular matrix or energy fluctuations will affect the status of matter of the same matrix (gelation and solation), where the water present behaves like a liquid crystal [31]. The “electrified” ions of the matrix will influence the speed of the fluids (electro-osmosis); the speed does not depend on valves or pumps but only on the electrical charge of the ions [32]. The biochemical reactions between the neurons and the cells that make up the nervous complex can create chemical waves under the aegis of the law of nonequilibrium thermodynamics, which would involve the centrosome in particular (MicroTubule Organizing Center - MTOC) [33]. The centrosome reorganizes the shape of the cell, changing the tension of the cell itself and varying the mechanical-electrical tension of the extracellular matrix, re-proposing the mechanism of gelation (gel or interconnected liquid phase) or of solation (sol or more solid colloidal suspension), influencing the velocity of fluids [33]. These mechanisms are not homogeneous in the central or peripheral nervous system and, therefore, are not able to influence the mobility of the nervous complex in its entirety. Some neurons can create nanotubes for the transport of mitochondria and other structural elements or molecules to another neuron or more neurons, varying the electro-mechanical voltage of brain areas, changing the speed status of brain fluids [34]. But, still, there is no homogeneity in the entire nervous system, and it should be emphasized that the existence of nanotubes lasts from a few minutes to hours [34]. According to the principle of quantum entanglement, all cells are in communication with each other, as a sort of wiring or ephaptic transmission, but the movement produced (oscillation, vibration, morphological deformation) always depends on the adaptation of a brain region, with respect to another [34-35]. These mechanisms are not able to move the central and peripheral nervous systems in unison. Another force that acts on the nervous system is vasomotricity, controlled by the sympathetic and parasympathetic nervous system and by the same structure of the contractile cells that form the vessel, the latter influenced by metabolic events. According to an osteopathic scientific view, the change in the tone of blood (and lymphatic) vessels would be at the basis of the cranial rhythm [8]. The oscillations are related to blood pressure and the different neurofluids, which are historically but inconsistently linked to measurable waves. We know the Traube-Hering waves (0.1 Hz) linked to blood pressure but, in reality, they do not reflect the vitality of the cranial rhythm that the osteopath feels with the manual evaluation, as 0.1 Hz corresponds to oscillations of pathology (loss of autonomic self-regulation) [36]. Another wider oscillation (0.005-2 Hz) is influenced by CSF in the subarachnoid space, but we know that the displacement of the CSF is heterogeneous and cannot represent a homogeneous rhythm such as that felt by palpation of the osteopath [36]. Furthermore, this oscillation is not equal between the two hemispheres and can be influenced by the patient's emotional status [36]. Another known and measurable oscillation are those of Mayer (0.1 Hz), but its nature remains not understood and doubtful; these oscillations are probably related to autonomic nervous activity and the displacements of interstitial fluids [37]. Mayer oscillations can also be recorded in the spinal cord, probably with the same reasons hypothesized for the central nervous system [38]. A mechanism capable of homogeneously moving the central nervous system and the spinal nervous system is that linked to the heart and the diaphragm muscle. We know that the central nervous system and spinal cord move 2-3 mL, stimulated by the respiratory diaphragm and the heartbeat. During systole, the nervous system is tractioned caudally and medially; the opposite occurs during diastole [8]. During an inhalation, the nervous system is pushed towards the cranially, while during an exhalation, the predominant movement is caudally [8]. The cardiovascular and respiratory systems work in perfect balance in a healthy subject and are controlled by the same neurological (and metabolic) systems: vagal system, sympathetic system, phrenic system [39]. The heart rate varies with the rhythm of breathing, just as the respiratory rate is affected by the behavior of the myocardium [39]. The mechanism of primary respiration (movement), according to the most up-to-date scientific information, cannot originate directly from the brain mass and spinal cord, but is a response (movement) determined by the breath/heartbeat. In the field of OCMM, it would be more accurate to talk about the secondary respiratory mechanism (SRM). Probably, when the osteopath places his hands on the skull for palpatory listening, he is primarily aware of the health of the cardiorespiratory system.

The mobility of the cranial and spinal meningeal membranes

**Figure 3:**
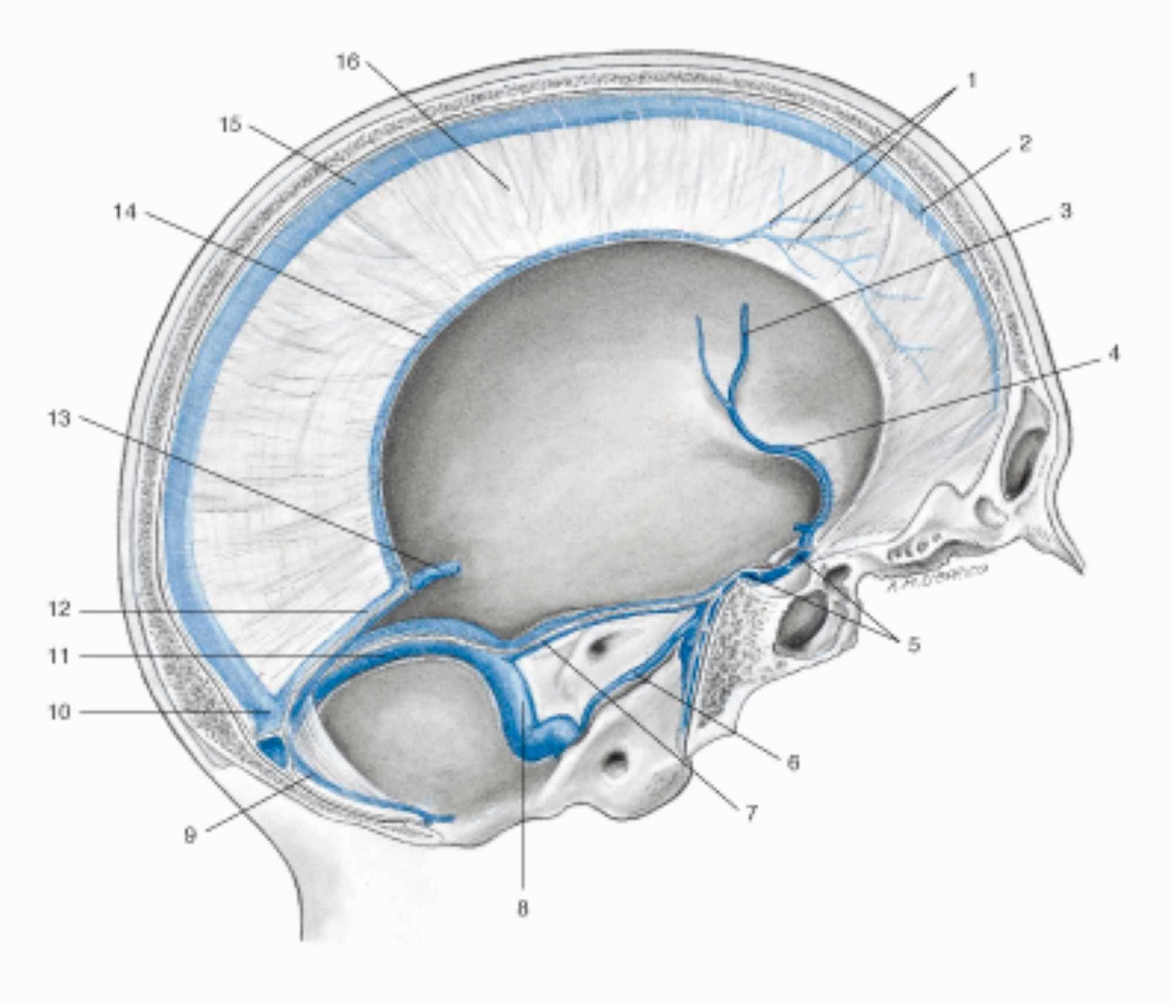
Representation of the venous sinuses of the dura mater in a sagittal section of the skull. 1 Veins of the dura mater; 2 Superior sagittal sinus; 3 Left middle cerebral vein; 4 Left sinus sphenoparietal; 5 Intercavernous sinus; 6 Left inferior petrosal sinus; 7 Left superior petrosal sinus; 8 Left sigmoid sinus; 9 Occipital sinus; 10 Confluence of sinuses; 11 Left transverse sinus; 12 Straight sinus; 13 Great cerebral vein (of Galen); 14 Inferior sagittal sinus; 15 Superior sagittal sinus; 16 Falx cerebri. Image reproduced with permission, from Anastasi G, et al., Anatomia dell'uomo, fourth edition [Human Anatomy], 2010, Milan: Edi-Ermes, Volume 1, Figure 4.145, p. 432.

The cranial meninges are the falx of the brain, the tentorium of the cerebellum, the falx of the cerebellum and the pituitary tent [7-8]. The dura mater of the skull, which covers the arachnoid layer and has an average thickness of one millimeter, has anisotropic viscoelastic properties, with a capacity to withstand mechanical forces of about 9-10 Pa (MPa which in this case is equivalent to 1 kilo per millimeter of square meter) [40]. The dura mater is defined as pachymeninge, while the arachnoid and the pial layer fall within the classification of leptomenynx. The meninges involving the area of the caudal forebrain and midbrain have an ectodermal origin, while the remaining meninges derive from the mesoderm (like the vessels found in the meninges) [41]. More specifically, the dura mater derives from the mesoderm, while the pia and the arachnoid derive from the mesoderm and the ectoderm [41]. The outer layer of the dura mater or dural periosteal layer is in direct contact with the periosteum of the skull, with which it exchanges arterial vessels [41]. The arachnoid layer has a thickness of about 200 µm; pia is a single layer of cells or basement membrane, with a specialized extracellular matrix covering the brain parenchyma and the vessels that penetrate the brain [41]. If the arachnoid acts as a shock absorber and as a dispersive element of mechanical tension, the pia also responds to mechanical stress and nociceptive stimuli [42]. The meninges are innervated by the autonomic system (vagal and sympathetic system), by cranial nerves (IV, V, VII, IX, XII) and by the first four cervical roots [7-8]. The meninges are important for venous outflow, from the dura mater with the venous sinuses, up to the pia with the bridging veins (the latter connected to the dural sinuses), passing through the arachnoid layer (Figure 3) [43].

The meningeal system is rich in lymphatic vessels, which, from a developmental point of view, is formed only after birth; they follow the course of the venous sinuses and the course of some arteries, such as the middle meningeal artery [44]. The meninges transmit mechanical forces from the outside to the inside (trauma), and from the inside to the outside; moreover, they are structures that reflect the mechano-metabolic variables of the environment in which they reside, changing their ability to manage tensions and varying their intrinsic structure over time [8]. With advancing age, the dura mater tends to ossify, particularly at the level of the sinuses of the dura mater, and this will affect the transmission of mechanical forces [8]. The folding of the dura mater to form the venous sinuses follows the morphology of the brain, which is never perfectly the same in the two hemispheres; it follows that the sinuses can be of a specific orientation (right or left), with an anisotropic tissue stiffness [8]. We do not have detailed data on meningeal behavior on the mode of transmission of the sensed tensions, other than the fact that they act as shock absorbers and that they can widen or reduce the propagation of tension (pressures). Looking at the studies on how the tissues surrounding the brain behave in the presence of trauma, it is clear that if the force is external and involves the skull, the tension produced is amplified; from the skin to the parenchyma, the different layers increase the force of pressure towards the brain [45]. We could assume, looking at the current data, that if a tension (pressure or traction) comes from the brain towards the skin, the different layers should dampen and slow down the propagation speed of the tension forces produced [45]. In fact, if a stress vector crosses layers with a significant stiffness (bone), the speed of the propagation waves will increase (from the outside to the inside); conversely, if the forces are produced from within, such as when the brain and spinal cord move influenced by breathing and heartbeat, the speed of propagation of the pressures created will be slower, as the internal tissues have less stiffness [46]. Probably, the cranial rhythm felt by the clinician's palpation derives from the movement of the brain and the spinal cord, which movement would create outward tension waves slower, compared to the real rhythm of breathing and heartbeat, thanks to the viscoelastic property of the meninges that dampen the speed of tension transmission. The spinal dura mater, which has a mesodermal embryological origin, envelops the spinal cord in its entirety; when the neurofluids cross the area protected by the dura mater, the latter stretches and presses against the vertebral area [47]. The cranial dura mater continues in the spinal cord, from the foramen magnum to the vertebral periosteal area; at the level of the second sacral vertebra (S2) it continues with a filiform structure, known as filum terminale, which connects the dural sac to the sacrum (periosteum) [47]. In the subdural space there are structures that stabilize the relationship of the dura with the spinal cord (fibrous septum posticum); the spinal dura is innervated in particular at the ventral level and less at the dorsal level, through the sinuvertebral nerves and by branches of the sympathetic system [47]. The dural spine is built to better resist longitudinal tensions and to a lesser extent lateral tensions. The internal dural layer is in continuity with the arachnoid one; the subarachnoidal space is symmetrical on the sides, but presents asymmetries between the ventral and dorsal area [47]. The dural sac can move in the longitudinal direction of 2-4 mm, a measure which coincides with the movement of the nervous system [47]. The spinal lymphatic system is found in the epidural space, together with adipose tissue [48]. The pial spinal layer has a great capacity to withstand longitudinal mechanical stresses and their distributions, despite its small thickness (from 0.089 to 1.40 MPa) [49-50].

## Conclusions

The OCMM relies on palpation of the skull to assess and possibly resolve changes in movement, shape, and orientation of the cranial bones. The review of the article, divided into two parts, discusses recent data on the synthesis and movement of CSF, highlights the movement of the central nervous system and the mechanical characteristics of the cranial and spinal meninges. From the first part of the article it is possible to deduce new reflections, such as that of naming the PRM with the name of SRM, and the possibility that the movement perceived by the osteopath's hands placed on the skull, derives from the movement of the brain (thanks to the heart and respiratory diaphragm), filtered by the damping of the cranial meninges.
